# The Illustrated Scientific News

**Published:** 1881-05

**Authors:** 


*The Illustrated Scientific News* for May is before us, looking handsomer, if possible, than any of the preceding issues. Since its change of publishers last January, this magazine has improved with each succeeding number. The present issue of the *Illustrated Scientific News* is overflowing with handsome engravings and interesting and instructive matter.

Among the various subjects illustrated in this issue is a superb specimen of cut glass ware; an exhaustive article on asphaltum and its use in streets and pavements; a new and ingenious hand-car, shown in operation; a new steel steamer for use in shallow rivers; the new Jobert telescope, and an interesting paper on physics without apparatus, also fully illustrated.

Every number contains thirty-two pages full of engravings of novelties in science and the useful arts. To be had of all news dealers, or by mail of the publishers, Munn & Co., 37 Park Row, New York, at $1.50 per annum ; single copies 15 cents.

